# Analysis of the Incidence and Influencing Factors of Depression in the Acute Stage of Ischemic Stroke: A Retrospective Clinical Study

**DOI:** 10.1002/brb3.70483

**Published:** 2025-04-21

**Authors:** Xiao Zhou, Saquib Waheed, Xinyin Cao, Madiha Fatim, Xiaohong Fu, Shilong Deng, Chong Chen, Sudong Qi, Hao Sun, Ke Cheng, Libo Zhao, Changlong Zhou

**Affiliations:** ^1^ Department of Neurosurgery, Yongchuan Hospital Chongqing Medical University Chongqing China; ^2^ Chongqing Key Laboratory of Cerebrovascular Disease Research Chongqing China; ^3^ NHC Key Laboratory of Diagnosis and Treatment on Brain Functional Diseases Chongqing China; ^4^ Yongchuan Hospital Chongqing Medical University Chongqing China; ^5^ Department of Neurology Yongchuan Hospital of Chongqing Medical University Chongqing China

**Keywords:** imaging, ischemic stroke, poststroke depression, risk factors

## Abstract

**Background:**

Poststroke depression (PSD) is a common complication following a stroke, but the risk factors for its onset remain controversial. The purpose of this study was to investigate the incidence of PSD and its relationship with stroke sites to provide more evidence for the early identification of high‐risk patients with PSD.

**Methods:**

This retrospective clinical study recruited acute ischemic stroke patients and assessed them for 2 weeks after the onset. Blood samples were collected from the patients upon admission for routine blood tests and blood biochemical analysis. Stroke patients with the Diagnostic and Statistical Manual of Mental Disorders 5th edition (DSM‐V) depressive diagnosis were rated for severity using the Hamilton Depression Rating Scale (HAMD), as measured by the National Institutes of Health Stroke Scale (NIHSS). Stroke prognosis was measured by the Modified Rankin Scale (mRS).

**Results:**

A total of 192 stroke patients were evaluated. Two weeks after the stroke, 73 patients developed depression, and the incidence of PSD was 38.02%. The proportion of depression composition after 2 weeks was as follows: 63 cases were mild depression, accounting for 91.8%; 6 cases were moderate depression, accounting for 8.2%. In univariate analysis, red blood cell (RBC) count, thalamic infarction, mRS score, and mini‐mental state examination (MMSE) score were identified as risk factors associated with the occurrence of PSD. Multivariate logistic regression analysis further confirmed that RBC count, mRS score, and MMSE score were significantly correlated with PSD development.

**Conclusion:**

This study suggests that patients with thalamic infarction and TOAST type I stroke should receive increased clinical attention. RBC count, high mRS scores, and high MMSE scores are three independent risk factors for PSD occurrence.

## Graphical abstract

Our study suggests that patients with thalamic infarction and TOAST type I stroke deserve more clinical attention. Red blood cell count, high mRS score, and high MMSE score are the three independent risk factors for the development of poststroke depression.

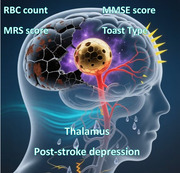

## 1 Introduction

Stroke is the second leading cause of death worldwide 2018. Poststroke depression (PSD), the most common neuropsychiatric sequela among stroke survivors, affects nearly 30%–50% of patients within the first year after stroke onset (Alexopoulos et al. 2002; Aström et al. 1993; Whyte et al. 2004). PSD can be categorized into acute (<1 month), subacute (1–6 months), and chronic phases (>6 months), with clinical manifestations including fatigue, apathy (Carnes‐Vendrell et al. 2019), sleep disturbances, anhedonia, pessimism, and cognitive impairment (Sachdev 2018). Key contributing factors to PSD include genetic predisposition (Igata et al. 2017; Levada and Troyan 2018; Liu and Gutierrez 2020), advanced age (Cheng et al. 2018), history of depression (Putaala 2020), and stroke severity (Hackett and Anderson 2005). However, its underlying mechanisms remain incompletely understood. Due to frequent comorbid cognitive and language impairments in stroke patients, PSD is often overlooked, significantly hindering rehabilitation. Studies highlight PSD as a critical risk factor for long‐term adverse physical and mental health outcomes (Schöttke et al. 2020), correlating with reduced quality of life and increased mortality (Sachdev 2018). Early identification and intervention for high‐risk PSD patients are thus crucial for recovery. Current research identifies elevated inflammatory markers (Yang et al. 2022), prior stroke history (Chaudhary et al. 2022), high serum leptin levels (Lee et al. 2015), and other factors as high‐risk indicators for PSD, whereas factors such as gender remain controversial (Cheng et al. 2018; Kutlubaev and Hackett 2014). Additionally, stroke lesion location may influence PSD risk, with evidence suggesting a higher incidence in patients with frontal lobe infarction (Nickel and Thomalla 2017; Rajashekaran et al. 2013). This study aims to analyze further risk factors associated with PSD during its acute phase, providing data to support clinical identification of high‐risk patients and early intervention strategies.

## 2 Subjects and Methods

### 2.1 Methods

This research was conducted at Yongchuan Hospital Affiliated to Chongqing Medical University. Patients diagnosed with stroke were enrolled in this study for 21 months, from April 2023 to January 2025. Computed tomography and/or magnetic resonance imaging supported stroke diagnosis for each patient.

### 2.2 Inclusion and Exclusion Criteria

The following were the requirements for patients with PSD to be included: age ≥18 years; stroke was limited to ischemic stroke (cerebral infarction), in line with the diagnostic criteria established in the Chinese guidelines for the diagnosis and treatment of ischemic stroke (2018 Edition) and confirmed by cranial CT or MRI; admitted to the hospital within 48 h after the onset of stroke; meeting the depression diagnostic criteria of Diagnostic and Statistical Manual of Mental Disorders 5th edition (DSM‐V); obtaining written consent from the patient or their legal representative. The exclusion criteria were as follows: patients receiving stroke‐related treatment outside the hospital; previously diagnosed with depression and other mental disorders or taking antidepressants and other psychotropic drugs; previously diagnosed with neurological diseases; having a history of brain trauma and brain surgeries; having severe infectious diseases, severe heart failure, and other severe chronic diseases; being pregnant or lactating women; complete aphasia, sensory aphasia, or apraxia hindering depression‐related assessment. The research protocol was approved by the Medical Ethics Committee of Yongchuan Hospital Affiliated with Chongqing Medical University (Appendix 1), and patients gave informed consent or their legal representatives.

### 2.3 Data Collection

For each patient, the following information was collected: baseline demographic data (age, gender, height, weight, smoking history, drinking history, history of hypertension, diabetes, hyperlipidemia, coronary heart disease, atrial fibrillation, history of stroke, medication history, etc.), laboratory test indicators (blood routine, biochemistry), imaging data, the National Institutes of Health Stroke Scale (NIHSS) measurement of stroke severity, the Oxford Community Stroke Project Classification of stroke type (TOAST classification), and the Modified Rankin Scale (mRS) measurement of stroke prognosis (outpatient follow‐up for discharged patients 2 weeks later).

### 2.4 Screening and Grading Tools for Depression

The Hamilton Depression Rating Scale (HAMD) was used to assess depressive symptoms in participants at the 2‐week mark. It was conducted by two trained professional neurologists and independently scored by two physicians. Participants with HAMD depression scores of 7 or more on two occasions were considered to have PSD. The intensity of depression was classified using the following criteria: scores below 6: no mood disturbance/normal; 7–17: mild depression; 18–24: moderate depression; 25 or above: severe depression.

### 2.5 Statistical Analysis for Comparisons Between the Two Groups

Continuous numerical variables conforming to a normal distribution were analyzed using the independent samples *t*‐test and expressed as mean ± standard deviation (SD). Non‐normally distributed continuous variables and ordinal categorical data were analyzed using the Mann–Whitney *U* nonparametric test, presented as median (interquartile range [IQR]; 25th–75th percentiles). Categorical data were assessed using the chi‐square test (Pearson or Fisher's exact test) and reported as percentages (%). Variables showing statistical significance in univariate analysis were included in a multivariate logistic regression model to identify independent risk factors for PSD. All statistical analyses were performed using SPSS 25.0.

## 3 Result

### 3.1 Incidence of PSD in 192 Stroke Patients

In this study, 192 stroke patients were enrolled. At 2 weeks’ poststroke, 73 patients developed depression, resulting in a PSD incidence rate of 38.02%. The distribution of depression severity at 2 weeks was as follows: 63 cases (91.8%) of mild depression, 6 cases (8.2%) of moderate depression, and 0 cases of severe depression. The clinical baseline characteristics included gender, age, height, weight, stroke type, and medical history (Table 1). Univariate analysis revealed a statistically significant difference in TOAST classification (*p* = 0.048). Regarding the NIHSS scores, mRS scores, and mini‐mental state examination (MMSE) scores (Table 2), patients with PSD exhibited significantly higher mRS scores (*p* < 0.01) compared to non‐PSD stroke patients, suggesting that severe poststroke physical disability is associated with a higher risk of PSD. Conversely, PSD patients had significantly lower MMSE scores (*p* < 0.001) than non‐PSD patients, indicating more pronounced cognitive impairment.

**TABLE 1:** Clinical characteristics of patients with and without poststroke depression.

Variables	PSD (*n* = 73)	Non‐PSD (*n* = 119)	
	*N* (%)	*N* (%)	*p* value
Gender			
Male	43 (58.9)	78 (65.55)	0.3607
Female	30 (41.1)	41 (34.45)	
Past history of stroke	8 (10.96)	11 (9.24)	0.8044
CVD risk factors			
Diabetes mellitus	17 (23.29)	29 (24.37)	0.8646
Smoking	25 (34.25)	49 (41.17)	0.3628
History of drinking	21 (28.77)	39 (32.77)	0.6316
Hypertension	47 (64.38)	76 (63.87)	>0.9999
Coronary heart disease	4 (5.48)	13 (10.92)	0.2964
Toast type			
1	42 (57.53)	46 (38.66)	0.048*
2	3 (4.11)	13 (10.92)	
3	21 (28.77)	44 (36.97)	
5	6 (8.22)	5 (4.20)	
	Median (IQR)	Median (IQR)	
Age (mean), years	71.37 (64.5–79.0)	68.52 (60–77)	0.0972
Height	1.607 (1.55–1.67)	1.607 (1.55–1.65)	0.9809
Weight	63.09 (55.0–70.0)	62.39 (57.0–70.0)	0.6582
Bmi	24.4 (22.23–26.95)	24.07 (21.78–27.67)	0.5105
Hospital stay	11.32 (8–13.5)	11.51 (7–15)	0.6817

*Note*: Values are expressed as number (%) or median (IQR): *indicates statistical significance.

Abbreviations: CVD, cerebral vascular disease; IQR, interquartile range; PSD, poststroke depression.

**TABLE 2:** National Institutes of Health Stroke Scale (NIHSS) score, Modified Rankin Scale (mRS) score, and mini‐mental state examination (MMSE) score.

	PSD (*n* = 73)	Non‐PSD (*n* = 119)	
Variables	Median (IQR)	Median (IQR)	*p* value
NIHSS score	3 (2–5.5)	3 (1–7)	0.7612
mRS score	3 (1–4)	2 (1–4)	0.0067**
Glasgow coma scale	15 (15–15)	15 (15–15)	0.7078
MMSE score	25 (20–27)	27 (23–29)	0.0001***

Abbreviations: IQR, interquartile range; PSD, poststroke depression.Blood samples were collected from the patients upon admission for routine blood tests and blood biochemical analysis.

*:Indicates statistical significance. *P < 0.05 for multivariable regression mode; **P < 0.01 for multivariable regression mode; ***P < 0.001 for multivariable regression mode.

### 3.2 Laboratory Findings

Among the laboratory findings (Table 3), the red blood cell (RBC) count showed a statistically significant difference between the two groups (*p* = 0.0368). The mean RBC count in the PSD group was 4.366 × 10^12^/L, compared to 4.566 × 10^12^/L in the non‐PSD stroke group. No significant abnormalities were observed in other laboratory parameters.

**TABLE 3:** Common relevant test indicators.

	PSD (*n* = 73)	Non‐PSD (*n* = 119)	
Variables	Mean ± SD	Mean ± SD	*p* value
Blood routine examination			
RBC	4.366(±0.6595)	4.566(±0.5659)	0.0368*
Hb	137.2(±21.00)	137.7(±17.31)	0.8686
HCT	40.36(±5.919)	42.21(±6.432)	0.0951
WBC	7.247(±2.458)	7.762(±2.631)	0.099
NEUT%	68.15(±13.32)	70.05(±14.32)	0.3845
EOS%	1.864(±3.445)	1.63(±2.160)	0.9358
LYM%	18.78(±10.70)	19.03(±11.12)	0.8799
MONO%	6.165(±2.319)	5.568(±2.001)	0.0614
PLT	207.5(±59.92)	213.5(±57.70)	0.2252
Glu	5.88(±1.638)	7.014(±3.672)	0.3075
Crea	77.5(±110.7)	75.48(±117.8)	0.7291
UA	330.4(±2.163)	340.1(±1.638)	0.9231
Urea	6.116(±1.638)	5.817(±2.085)	0.3082
TC	4.758(±1.079)	4.962(±1.12)	0.2648
TG	1.861(±1.232)	1.708(±0.8955)	0.7286
HDL	1.065(±0.2534)	1.077(±0.2477)	0.6195
LDL	2.517(±0.7513)	2.791(±0.8176)	0.0516
ALT	20.69(±9.956)	20.72(±12.52)	0.5861
AST	25.57(±8.859)	25.94(±9.228)	0.8166
K	3.821(±0.4687)	3.873(±0.4401)	0.4204
Na	140.2(±3.515)	140.5(±3.53)	0.8153
Cl	104.8(±3.823)	105.1(±4.198)	0.838

Abbreviations: ALT, alanine aminotransferase; AST, aspartate aminotransferase; Cl, chloride; Crea, creatinine; EOS%, eosinophil percentage; Glu, glucose; Hb, hemoglobin; HCT, hematocrit; HDL, high‐density lipoprotein; K, potassium; LDL, low‐density lipoprotein; LYM%, lymphocyte percentage; MONO%, monocyte percentage; Na, sodium; NEUT%, neutrophil percentage; PLT, platelet; PSD, poststroke depression; RBC, red blood cell; SD, standard deviation; TC, total cholesterol; TG, triglyceride; UA, uric acid; WBC, white blood cell.

*:Indicates statistical significance. *P < 0.05 for multivariable regression mode; **P < 0.01 for multivariable regression mode; ***P < 0.001 for multivariable regression mode.

### 3.3 Imaging Analysis

In the imaging data analysis, five PSD patients and seven stroke patients were excluded from statistical evaluation because they underwent initial imaging at external hospitals and were subsequently transferred to our institution for continued care. No significant differences were observed in the imaging characteristics between the groups (Table S1). Further analysis of infarction sites stratified by left/right hemisphere localization revealed that thalamic infarction was identified as a risk factor for PSD (Table 4). At the same time, no significant differences were observed in infarctions at other locations.

**TABLE 4:** Imaging data.

Position		PSD (*n* = 68)	Stroke (*n* = 112)	*p* value
Frontal cortex	No	38	68	0.808
	Left	12	14	
	Right	13	21	
	Both	5	9	
Temporal cortex	No	56	86	0.673
	Left	4	9	
	Right	8	17	
	Both	0	0	
Occipital cortex	No	56	89	0.869
	Left	4	5	
	Right	7	16	
	Both	1	2	
Parietal cortex	No	52	77	0.179
	Left	8	9	
	Right	5	21	
	Both	3	5	
Paraventricular	No	40	79	0.228
	Left	5	10	
	Right	14	16	
	Both	9	7	
Half oval center	No	56	101	0.428
	Left	3	3	
	Right	5	5	
	Both	4	3	
Basal ganglia	No	44	76	0.667
	Left	9	12	
	Right	11	21	
	Both	4	3	
Thalamus	No	61	103	0.009**
	Left	3	3	
	Right	0	6	
	Both	4	0	
Corpus callosum	No	66	111	0.141
	Left	0	0	
	Right	2	0	
	Both	0	1	
Brainstem	No	62	104	0.286
	Left	2	3	
	Right	4	2	
	Both	0	3	
Cerebellum	No	59	96	0.954
	Left	2	6	
	Right	5	7	
	Both	2	3	

*:Indicates statistical significance. *P < 0.05 for multivariable regression mode; **P < 0.01 for multivariable regression mode; ***P < 0.001 for multivariable regression mode.

### 3.4 Multivariate Logistic Regression Analysis

A multivariate logistic regression analysis was performed on the identified risk factors from the study, which revealed that mRS scores, MMSE scores, and RBC count were independent risk factors for PSD occurrence (Table 5).

**TABLE 5:** Logistic regression analyses for predictive variables.

Factors	β	SE	Wald	df	*p*	OR	95% CI
Toast type	0.141	0.139	1.02	1	0.313	1.151	0.876–1.513
mRS score	−0.236	0.115	4.225	1	0.04*	0.789	0.63–0.989
MMSE score	0.104	0.03	11.721	1	0.001**	1.109	1.045–1.177
Thalamus	−0.292	0.262	1.246	1	0.264	0.746	0.447–1.247
RBC	0.653	0.275	5.625	1	0.018*	1.921	1.12–3.294
Constant	−4.619	1.626	8.072	1	0.004	0.01	

Abbreviations: MMSE, mini‐mental state examination; mRS, modified rankin scale; RBC, red blood cell.

*p < 0.05 for multivariable regression mode.

**p < 0.01 for multivariable regression mode.

## 4 Discussion

PSD, as one of the most common complications following stroke, is insidious in onset and imposes a substantial burden on patients, families, and society. Early detection and diagnosis of PSD are critical to facilitate patient recovery and alleviate societal and familial burdens. This study focused exclusively on ischemic stroke. A meta‐analysis suggests that the association between depression and stroke may not differ significantly across stroke subtypes (hemorrhagic vs. ischemic) (Sachdev 2018).

The incidence of PSD in our cohort was 38.02%, consistent with previously reported rates (Alexopoulos et al. 2002; Aström et al. 1993). Notably, this study specifically addressed acute PSD, whereas chronic PSD remains a significant concern. A UK‐based survey reported a PSD incidence of 28% during the acute phase, rising to 56% in the chronic phase (Camões Barbosa et al. 2011; Haq et al. 2010). This disparity may stem from prolonged socio‐familial stressors that hinder patient adaptation poststroke. Adequate family support and social engagement may mitigate the severity and incidence of chronic PSD.

In this study, the mean age of PSD patients was 71.37 years, slightly higher than that of non‐PSD stroke patients (68.52 years), though this difference was not statistically significant (*p* = 0.0972). The role of age as a risk factor for PSD remains debated: Some studies report no association (Putaala 2020), whereas others identify age as a contributing factor (Hackett and Anderson 2005). Gender may also influence PSD risk. In our cohort, the incidence of PSD was 35.5% in males and 42.3% in females. However, conflicting evidence suggests that males may be more vulnerable due to heightened familial and societal pressures (Kulkantrakorn and Jirapramukpitak 2007).

The TOAST classification, closely linked to stroke severity (Flach et al. 2020; Swanepoel and Pretorius 2015; Wang et al. 2022), showed significant differences between the two groups (*p* = 0.048). The proportion of TOAST type I (large artery atherosclerosis) was markedly higher in the PSD group (57.53%) compared to the non‐PSD group (38.66%) (*p* = 0.0118, <0.05) (Table 6), underscoring the need for heightened clinical attention to patients with this stroke subtype.

**TABLE 6:** TOAST type I analyses.

Factor	PSD (*n* = 73)	Stroke (*n* = 119)	*p* value
TOAST type I			
Yes	42	46	0.0118*
No	31	73	

Abbreviation: PSD, poststroke depression.

*p < 0.05 for multivariable regression mode.

Among the NIHSS score, mRS score, Glasgow Coma Scale, and MMSE score, the mean mRS score was significantly higher in the PSD group (2.63) compared to the stroke‐only group (2.05), indicating greater disability and poorer prognosis in PSD patients. Studies confirm that moderate‐to‐severe disability increases the risk of PSD by approximately 20% (Naess et al. 2005) and elevates mortality risk (Graber et al. 2020), with this phenomenon being more pronounced in younger populations (Naess et al. 2005; Zhang et al. 2013). The MMSE score was also lower in the PSD group (22.63) than in the stroke‐only group (25.42), suggesting more severe cognitive dysfunction in PSD patients. Severe cognitive impairment may reduce daily functional capacity and exacerbate depressive symptoms, aligning with prior research (Roberts et al. 2024).

Regarding routine laboratory parameters, the PSD group exhibited a lower RBC count (4.366 × 10^12^/L) compared to the stroke‐only group (4.566 × 10^12^/L) (*p* < 0.05), though both values remained within normal ranges. Although studies suggest a potential association between red cell distribution width (RDW) and PSD (Li et al. 2022; Wang et al. 2020), the relationship between RBC count and PSD requires further validation. Inflammation is implicated in PSD pathogenesis (Chi et al. 2021; Zhang et al. 2024), with Xiao demonstrating that innate immune‐mediated neuroinflammation promotes PSD development (Xiao et al. 2024). A 2022 meta‐analysis highlighted elevated C‐reactive protein (CRP) levels during the acute stroke phase as a predictor of PSD (Yang et al. 2022). In our study, the PSD group showed marginally lower white blood cell (WBC) counts (7.247 vs. 7.762, *p* = 0.099) and higher monocyte levels (6.165% vs. 5.568%, *p* = 0.0614). However, no significant differences in other inflammatory markers were observed (Table S2). Nonetheless, inflammation undeniably plays a critical role in PSD.

In imaging analysis, thalamic infarction was strongly associated with PSD. The thalamus integrates sensory pathways and projects to the cerebral cortex, playing a vital role in daily functioning. Yang reported that left thalamic lesions correlate closely with poststroke depressive symptoms (Fritsch et al. 2021), supporting our findings. In hemorrhagic stroke, thalamic hemorrhage has been linked to anxiety‐depressive behaviors (Infantino et al. 2022; Shi et al. 2023). A recent study identified right thalamic infarction as a contributor to poststroke cognitive impairment (Mu and Li 2024), which may exacerbate PSD severity (Baccaro et al. 2019; Perrain et al. 2020), though this remains unreported in ischemic stroke. Although prior studies associate frontal lobe lesions, particularly left frontal involvement, with PSD (Nickel and Thomalla 2017; Stein et al. 2018), no such correlation was observed in our cohort.

Our study has several limitations. First, it is a single‐center investigation with a relatively small sample size (*n* = 192), and all included patients were of Han Chinese ethnicity. Prior research suggests that race/ethnicity may influence PSD risk (Glymour et al. 2012), highlighting the need for broader demographic representation. Future studies will focus on validating the association between thalamic infarction and PSD by collecting larger, multiethnic cohorts to minimize potential biases. Second, patients with aphasia or those unable to cooperate were excluded, though poststroke aphasia itself is linked to higher depression risk (Kao and Chan 2024), potentially skewing our findings. Additionally, pre‐stroke depression—a known PSD risk factor—was not adequately assessed. In China, limited healthcare access and low awareness of mental health among elderly populations often lead to underdiagnosis of pre‐existing depression. Studies report a 13.3% prevalence of major depression in older adults (Abdoli et al. 2022; Tan et al. 2023), a subgroup inherently more vulnerable to PSD.

## 5 Conclusion

In conclusion, our findings indicate that stroke patients with lower RBC counts, severe physical disability (high mRS scores), and cognitive deficits (low MMSE scores) are at heightened risk for PSD. Thalamic infarction and TOAST type I stroke emerged as critical predictors of PSD, warranting prioritized clinical attention and targeted interventions for these populations.

## Author Contributions

**Xiao Zhou**: methodology, data curation, formal analysis, writing–review and editing, writing–original draft, validation, visualization, resources, software. **Saquib Waheed**: conceptualization, investigation, writing–original draft, writing–review and editing, supervision, data curation. **Xinyin Cao**: data curation, resources, investigation, project administration. **Madiha Fatim**: writing–review and editing, data curation, formal analysis, validation. **Xiaohong Fu**: methodology, conceptualization, data curation, supervision. **Shilong Deng**: project administration, supervision. **Chong Chen**: supervision, project administration. **Sudong Qi**: supervision, project administration. **Hao Sun**: supervision, project administration. **Ke Cheng**: conceptualization, methodology, supervision, formal analysis. **Libo Zhao**: resources, project administration, methodology, data curation, supervision. **Changlong Zhou**: conceptualization, writing–review and editing, funding acquisition, project administration, resources, supervision, methodology.

## Consent

Publication of the manuscript has been approved by all coauthors.

## Conflicts of Interest

The authors declare no conflicts of interest.

### Peer Review

The peer review history for this article is available at https://publons.com/publon/10.1002/brb3.70483

## Supporting information



Supporting Information

## Data Availability

All data used to support the findings of this study are available from the corresponding author upon request.
