# Supplementary material for: Analysis of the Incidence and Influencing Factors of Depression in the Acute Stage of Ischemic Stroke: A Retrospective Clinical Study
Source: Brain Behav. 2025 Apr 21;15(4):e70483. doi: 10.1002/brb3.70483 (PMC12012248; doi:10.1002/brb3.70483)
Supplement: Supplementary file 1 — Supporting Information [file BRB3-15-e70483-s001.docx]

Appendix 1：Ethics approval


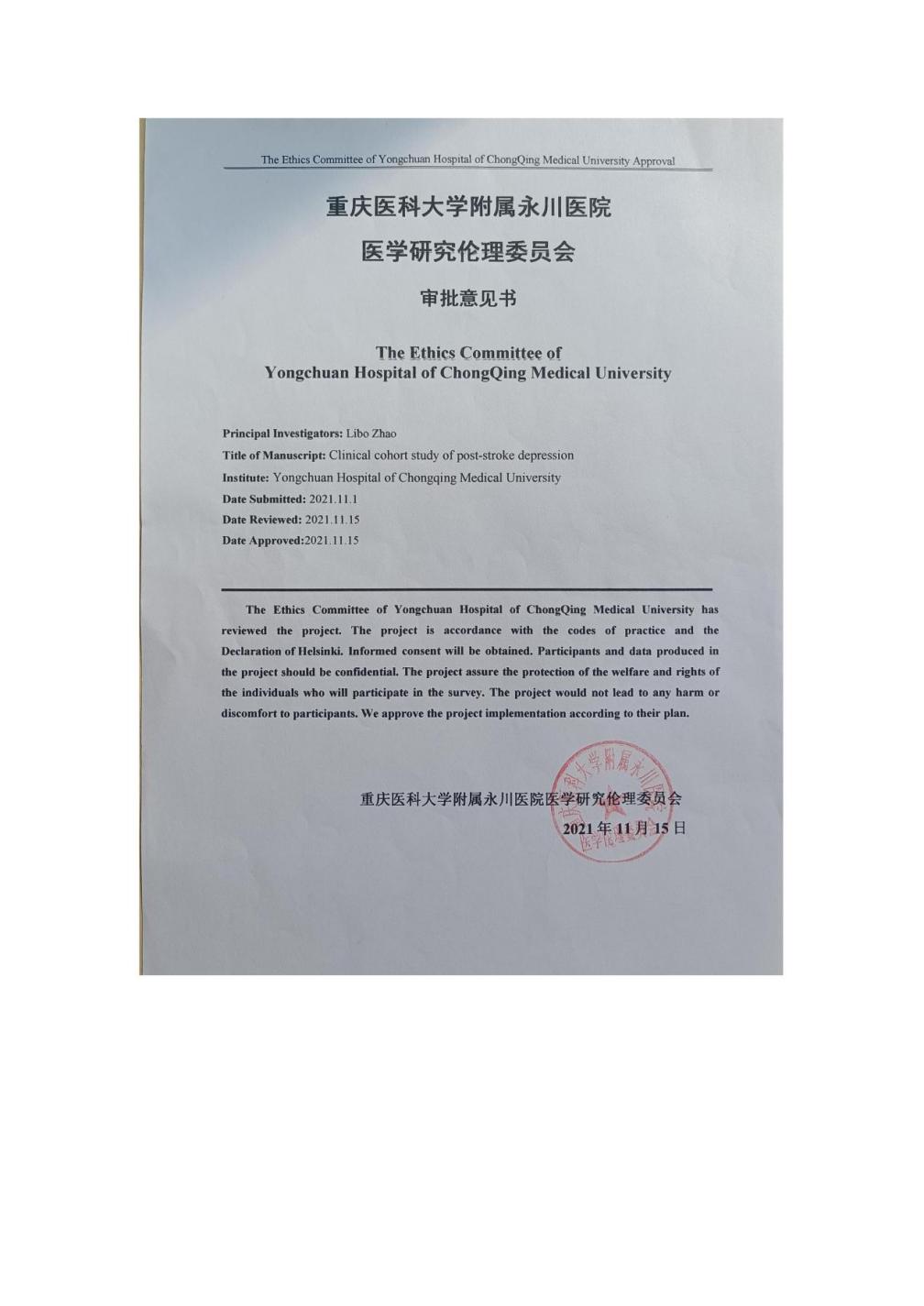


Supplementary Table 1

Imaging Data

| position |  | PSD（n=68） | Stroke（n=112） | P value |
| --- | --- | --- | --- | --- |
| Frontal cortex | no | 38 | 68 | 0.523 |
|  | yes | 30 | 44 |  |
| Temporal cortex | no | 56 | 86 | 0.4527 |
|  | yes | 12 | 26 |  |
| Occipital cortex | no | 56 | 89 | 0.7006 |
|  | yes | 12 | 23 |  |
| Parietal cortex | no | 52 | 77 | 0.308 |
|  | yes | 16 | 35 |  |
| Paraventricular | no | 40 | 79 | 0.1435 |
|  | yes | 28 | 33 |  |
| Half oval center | no | 56 | 101 | 0.0523 |
|  | yes | 12 | 11 |  |
| Basal ganglia | no | 44 | 76 | 0.1273 |
|  | yes | 24 | 36 |  |
| thalamus | no | 61 | 103 | 0.6007 |
|  | yes | 7 | 9 |  |
| Corpus callosum | no | 66 | 111 | 0.5578 |
|  | yes | 2 | 1 |  |
| brainstem | no | 62 | 104 | 0.7762 |
|  | yes | 6 | 8 |  |
| cerebellum | no | 59 | 96 | >0.9999 |
|  | yes | 9 | 16 |  |

Supplementary Table 2

Partial inflammatory indicators

| Factors | PSD(N=33) | STROKE(N=47) |  |
| --- | --- | --- | --- |
|  | Median (IQR) | Median (IQR) | P value |
| SII | 771(428,1073) | 768(439,1073) | 0.7119 |
| NLR | 3.31(2.21,5.57) | 3.36(2.12,6.71) | 0.7338 |
| PLR | 147（108,192） | 151（104,223） | 0.7265 |

SII：Systemic Immune-Inflammation Index；NLR：Neutrophil to Lymphocyte Ratio；PLR：Platelet to Lymphocyte Ratio
